# Impact evaluation of the Hypertension Treatment in Nigeria program using the Translational Science Benefits Model

**DOI:** 10.1017/cts.2026.10790

**Published:** 2026-07-13

**Authors:** Guhan Iyer, Nanna Ripiye, Ikechukwu Anthony Orji, Gabriel Lamkur Shedul, Grace J. Shedul, Eugenia N. Ugwuneji, Tunde M. Ojo, Emmanuel Okpetu, Samuel Osagie, Namratha Kandula, Malau M. Toma, Bunmi R. Oshundele, Sunday Victor Eze, Erica L. Jamro, Chisom Obiezu-Umeh, Abigail S. Baldridge, Lisa R. Hirschhorn, Dike Bevis Ojji, Mark D. Huffman

**Affiliations:** 1 Washington University in St Louis, St. Louis, MI, USA; 2 University of Abuja, Abuja, Nigeria; 3 Northwestern University Feinberg School of Medicine, Chicago, IL, USA; 4 Nigeria Federal Ministry of Health, Abuja, Nigeria; 5 Non-Communicable Diseases and Control Division, Nigeria Federal Ministry of Health and Social Welfare, Abuja, Nigeria; 6 The George Institute for Global Health, Sydney, NSW, Australia

**Keywords:** Hypertension, implementation research, impact evaluation, Nigeria

## Abstract

**Background:**

Hypertension is a leading cause of cardiovascular disease with significant burden experienced in low-and middle-income countries. The Hypertension Treatment in Nigeria (HTN) Program implemented a large-scale hypertension treatment program in the Federal Capital Territory (FCT) of Nigeria. Identification of benefits is important to acknowledge and demonstrate the value of research beyond evaluation of pre-identified scientific outcomes. This manuscript aims to identify the benefits and impact of the HTN Program using the Translational Science Benefits Model (TSBM).

**Methods:**

The HTN Program was a type II interrupted time series trial conducted from January 2020 to December 2023 to evaluate effectiveness and implementation of a multilevel hypertension control program in 60 primary healthcare centers in Nigeria. TSBM benefits were identified and verified by cataloging events, results, and procedures and aligning using the Translating for Impact Toolkit. The domains include policy, community, clinical, economic, and a new fifth domain, capacity building.

**Results:**

The HTN Program enrolled 21,922 patients. Hypertension treatment increased from 86.8% (95%CI: 86.5%, 87.2%) at baseline to 96.2% (95%CI: 96.0%, 96.4%) at the end of the study period. Hypertension control also increased from 22.3% (95%CI: 21.7%, 22.9%) at baseline to 56.4% (95%CI: 55.9%, 56.8%). Thirty-three TSBM benefits were identified across five domains. Most benefits were identified in the policy (9), capacity building (9), and community (7) domains.

**Conclusion:**

The HTN Program not only improved hypertension treatment and control in primary care in the FCT of Nigeria but also demonstrated broader impacts beyond its initial effectiveness and implementation objectives supporting program sustainment and scale-up.

Trial Registration: NCT04158154.

## Introduction

Hypertension is one of the leading causes of preventable death globally, contributing to an estimated 10.8 million deaths and 235 million disability-adjusted life years (DALYs) each year [1]. Similar to other non-communicable, chronic diseases (NCDs), this burden is unequally distributed, with low-and middle-income countries (LMICs) experiencing a higher relative and absolute burden of hypertension compared to high-income countries [2] Nigeria has an estimated hypertension prevalence of 33%. Of that group, only an estimated 47% receive a hypertension diagnosis, 27% receive treatment, and control rates are approximately 11% [1,3]. Despite the high burden of disease and major gaps in the cascade of care, research evaluating the implementation and effectiveness of multilevel strategies to improve hypertension care in primary healthcare settings remains limited in Nigeria and most other LMICs.

The Hypertension Treatment in Nigeria (HTN) Program was a type II hybrid effectiveness-implementation study that adapted, implemented, and evaluated a multilevel hypertension bundle modeled after the Kaiser Permanente Northern California (KPNC) Program and the World Health Organization (WHO) HEARTS technical package. The WHO HEARTS (Healthy-lifestyle counseling, Evidence-based treatment, Access to essential medicines, Risk-based treatment, Team-based care, Systems for monitoring) package is a globally recognized set of strategies designed to improve primary healthcare systems’ ability to provide hypertension care services [4]. Strategies include expanding the scope of work and healthcare worker privileges for trained and supervised non-physician healthcare workers, such as community health extension workers (CHEWs), ensuring readiness for hypertension evaluation and management, and improving supply chain and information systems.

The first phase of the program included recruiting 60 primary health centers (PHCs) in the Federal Capital Territory (FCT) of Nigeria. After evaluating service availability and readiness for hypertension and adaptation of the multilevel bundle, patient-level data were collected from January 2020 to December 2023. The primary aim of the HTN Program was to improve hypertension treatment and control in PHCs using an interrupted time series design (Ojji et al. under review) [5]. Implementation outcomes were also assessed using mixed methods guided by the Reach, Effectiveness, Adoption, Implementation, and Maintenance (RE-AIM) framework [6,7].

While the main research outcomes of the HTN Program focused on effectiveness and implementation outcomes, additional benefits have been identified using the Translational Science Benefits Model (TSBM), which was developed by the Institute of Clinical and Translational Science at Washington University [8]. This model offers a framework to define areas of impact that provide benefits to public health and the society that are typically not captured in traditional methods of determining clinical or implementation research or program “success” [8]. The TSBM contains four domains in which impact may be realized: (1) Clinical and Medical, (2) Community and Public Health, (3) Economic, and (4) Policy. Thirty benefit categories are distributed across these domains, representing areas of impact that can be achieved through the implementation of translational research [8]. The TSBM recognizes that impact occurs as a result of conducting the study. The TSBM has been extensively operationalized in the US, including in resource limited settings [9,10]. Ensuring accurate and timely reporting of “extra-scientific” impact can bolster the value proposition of translational research. While other impact frameworks exist, the TSBM generally has a higher degree of operationalization and granularity along with a user-friendly interface to catalog and sort benefits but previous use in LMIC settings is limited. The objective of this report is to use the TSBM to identify and show the benefits associated with the HTN Program and explore its efficacy in LMIC settings.

## Methods

The methods, primary implementation, and effectiveness outcomes of the HTN Program have been reported [5]. Briefly, key effectiveness outcomes for the HTN Program included differences in hypertension treatment and control, as well as systolic and diastolic blood pressure between the pre-implementation and implementation periods. Implementation outcomes not only included RE-AIM measures, but also measures of cost, normalization [11], and sustainability [12] as well as implementation of adjunctive strategies, including supportive supervision and drug revolving fund [13].

### Multilevel strategy bundle adapted from KPNC and WHO HEARTS

The components of the multilevel bundle are shown in Table 1. Briefly, the intervention comprised seven components adapted from the KPNC and WHO HEARTS models: Patient registry/empanelment (health system level), Performance and quality reporting (clinic level), Team-based care (health worker level), Access to essential medicines and technology (health system level), Fixed dose combination prioritization (health system level), Simplified treatment protocol (national policy level), and Health coaching and home blood pressure monitoring (patient level) [5]. A formative period was used to identify PHCs that met the inclusion criteria and to conduct a Service Availability and Readiness Assessment to determine each PHC’s capacity and readiness for adapting and implementing the proposed bundle [14,15]. The program also included implementation of a drug revolving fund and supportive supervision [13]. Additionally, more than 300 community engagement events were developed and implemented to raise hypertension awareness among populations served by the 60 PHCs based on recommendations of community leaders identified during formative evaluation [15]. Adults with persistently elevated blood pressure (≥140/90 mmHg measured on two occasions) or a history of hypertension were sequentially enrolled and empaneled at PHCs. Community leaders and practitioners with significant experience in the local context advised the research team on appropriate adaptation of the multilevel intervention to best align with the structural and cultural factors present in this population [15].

**Table 1.** Structural components of multilevel hypertension control programs compared to the Hypertension Treatment in Nigeria (HTN) Program Table 1 long description.

Component	Kaiser Permanente Northern California model	World Health Organization HEARTS model	Hypertension Treatment in Nigeria program
Patient registry/empanelment	Hypertension patient registration using baseline data collected in the formative period	Hypertension patient registration	Hypertension patient registration and longitudinal empanelment
Performance and quality reporting	Monthly quality and performance reports, including treatment and control rates to sites	Information systems that allow for continuous, real-time program improvement	Monthly automated quality and performance feedback reports. Real-time registry dashboard for patient follow-up
Simplified treatment protocols and patient care	Simplified treatment guidelines Use of fixed-dose combinations, such as lisinopril-hydrochlorothiazide as first-line treatment	Protocols that include specific medications, doses, and steps to streamline care and improve adherence and control Patient-centered care with simpler medication regimens, free or low-cost medications and follow-up visits, and readily available blood pressure monitoring	Simplified treatment guideline and national hypertension consensus protocol
Supply chain (including fixed-dose combination prioritization and access to essential medicines)	Use of fixed-dose combinations, such as lisinopril-hydrochlorothiazide as first-line treatment	Ensuring a reliable, uninterrupted supply of quality blood pressure lowering medicines	Supply chain strengthening through the Drug Revolving Fund for blood pressure lowering drug availability and affordability
Team-based care	Non-physician follow-up 2-4 weeks after medication changes	Use of non-physician health workers to prescribe, adjust, and intensify medication regimens based on physician orders and protocols	Empowerment and enablement of community health extension workers as first line for diagnosis, treatment and management of patients with hypertension
Supportive supervision	Not included	Not included	Implementation strategy to provide guidance, mentorship, and resource allocation to healthcare workers to improve performance

### TSBM structure

In addition to the four original TSBM domains, the HTN Program team included a fifth domain: “Capacity Building,” because a large portion of the program included training and supervising healthcare workers, expanding the scope of practice among healthcare workers, improving the PHC system’s capacity, and providing opportunities for career development among the members of the research team. These benefits are not currently included in the TSBM but align with the wider definition of research impact that the TSBM aims to identify and promote (personal communication: Professor Douglas Luke).

Translational impact identification was conducted between April 2024 and March 2025 using the online Translating for Impact Toolkit hosted by Washington University (https://translationalsciencebenefits.wustl.edu/toolkit/#/thetoolkit). The study team first compiled a descriptive inventory of HTN Program activities and observable outcomes (e.g., trainings delivered, policies implemented/proposed, community meetings conducted, etc.) from project documentation and team reports. Three team members mapped these activities and outcomes to the published TSBM domains and indicators using operational definitions. Items that did not fit clearly within existing domains were flagged for larger team review. Throughout this process, the study team noted a pattern of training and infrastructure development not captured by the original domains, motivating the inclusion of the novel Capacity Building domain. A distinction between demonstrated and potential benefits was implemented to clarify benefits that have been realized with those that have not. Benefits were classified as “demonstrated” when there was evidence the outcome had already taken place (e.g., passed policy changes or number of personnel trained, etc.). Potential benefits reflect plausible future outcomes for which evidence has not been observed (e.g., a policy bill under consideration with potential to pass or an enhanced healthcare delivery practice implemented in a local context but with potential to expand, etc.). Additionally, a distinction between process-and outcome-related benefits was included to identify benefits resulting from activities associated with implementation of the HTN Program compared to benefits resulting from outcomes of the HTN Program.

## Results

### Effectiveness outcomes of the HTN program

A total of 21,922 patients were enrolled in the HTN Program from January 2020 to December 2023. Over the study period, 142,492 clinic visits were recorded. Median (IQR) age was 49 (40, 58) years and 68.2% of patients were female (Ojji et al. under review). Using a six-month rolling average, baseline treatment increased from 86.8% (95% CI: 86.5%, 87.2%) at the start of the study period to 96.2% (95% CI: 96.0%, 96.4%) at the end of the study period. Baseline control increased from 22.3% (95% CI: 21.7%, 22.9%) to 56.4% (95% CI: 55.9%, 56.8%). Mean systolic blood pressure decreased from 152.3 mmHg (95% CI: 152.0, 152.5) at baseline to 135.8 mmHg (95% CI: 135.6, 135.9) at the end of the study. The program demonstrated wide reach, adoption, implementation fidelity, and maintenance, and measures of normalization and sustainability were high.

### TSBM benefits across domains

**Figure 1.**
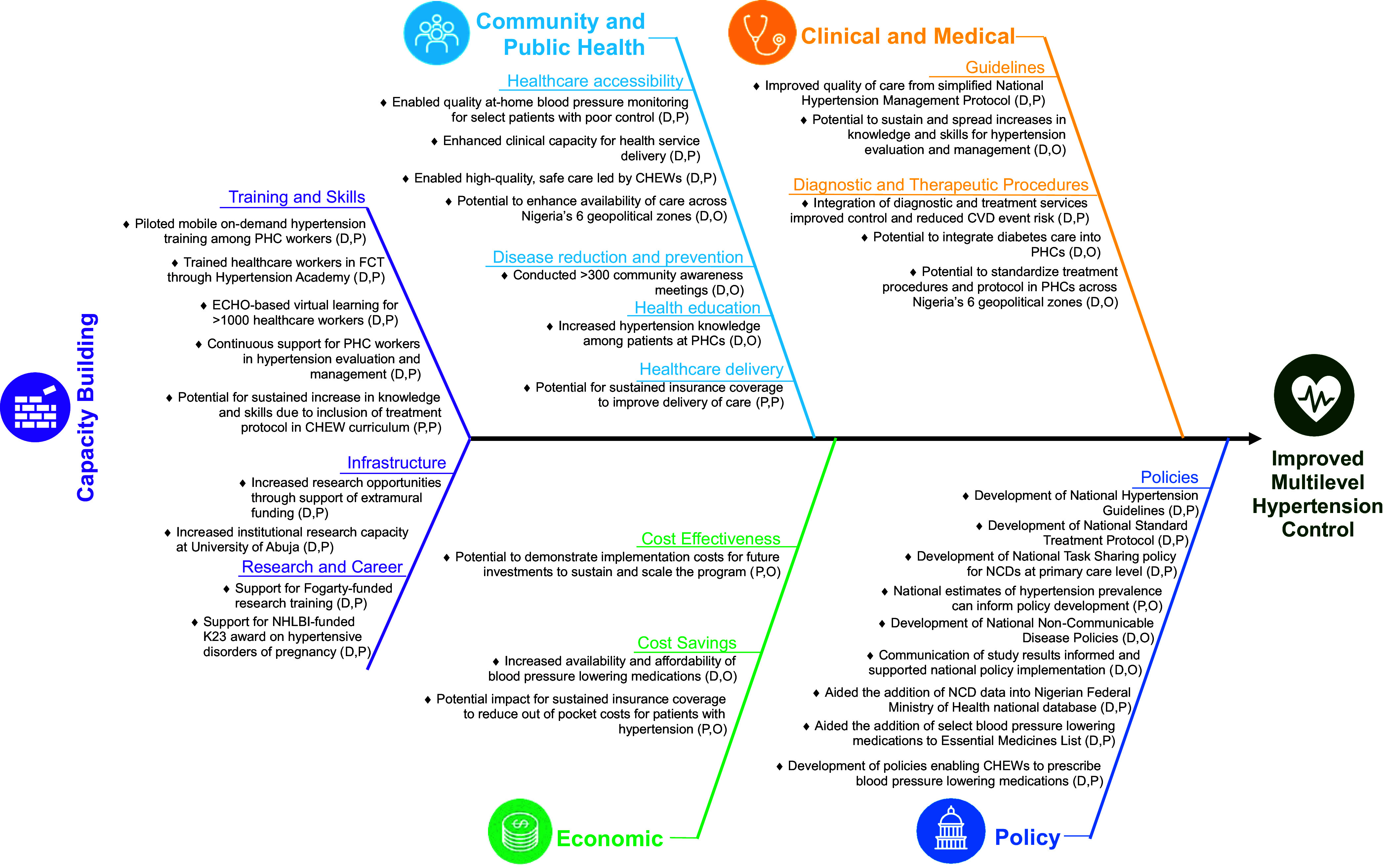
Identification of program activities and outcomes leading to TSBM benefits grouped by indicator, domain, (a) demonstrated (D) vs. potential (P) and (b) outcome (O) vs. process (P). TSBM = Translational Science Benefits Model.

Thirty three benefits (25 observed, 8 potential) were identified in five domains (Figure 1). The domains with the largest number of impact areas were “Policy” (9), “Capacity Building” (9), and “Community and Public Health” (7). Twenty-one benefits were perceived to be process-related, while the remaining 12 were outcome-related.

#### Policy

The demonstrated benefits within the Policy domain are a result of continuous collaboration and communication with municipal, state, and federal health entities in Nigeria. Eight identified benefits were demonstrated (one potential), and six benefits were classified as process-related. The three outcome-related benefits were a result of HTN Program findings being used to inform policy development and implementation, such as adding blood pressure lowering medications to the country’s essential medicines list, informing new task shifting and sharing policies, and contributing to national guidelines of economic support of primary health services (Figure 1) [16–18].

#### Capacity building

Capacity building activities revolved around training and skills, career development, and infrastructure improvement. The nine capacity building benefits extended beyond hypertension treatment to other NCDs, including diabetes. These benefits were grouped into three indicators: “Training and Skills,” “Infrastructure,” and “Research and Career Development.” The training of CHEWs to manage hypertension with the Hypertension Academy was an example of a benefit within the Training and Skills domain. Establishment and enhancement of research infrastructure at the University of Abuja (e.g., REDCap infrastructure) to appropriately capture and manage incoming data was an example of an Infrastructure benefit. Use of HTN Program infrastructure allowed early-stage investigators to pilot substudies, supporting subsequent grant applications and career development [19,20]. All benefits were identified as process-related benefits.

#### Community and public health

The seven identified benefits in the “Community and Public Health” domain were spread across four benefit indicators: (i) Healthcare Accessibility, (ii) Disease Reduction and Prevention, (iii) Health Education, and (iv) Health Delivery. These benefits largely stemmed from the improvement in healthcare delivery within communities at the PHC level through implementation of the HTN Program and from implementation of the community awareness events. Out of the seven identified benefits, five were considered demonstrated benefits (2 potential), and four were process-related benefits (3 outcome-related, Figure 1).

#### Clinical and medical

Demonstrated and potential benefits within the “Clinical and Medical” domain focused on improving the quality of hypertension management in routine primary care in the FCT through implementation of the HTN Program. The five identified benefits fell under two TSBM indicators: “Diagnostic and Therapeutic Procedures” and “Guidelines,” the latter which included national treatment protocol that was co-developed by HTN Program team members.

#### Economic

Three economic benefits were identified because of the HTN Program across two domains: “Cost Effectiveness” and “Cost Savings.” Implementation of the HTN Program increased availability and affordability (i.e., the two components of accessibility) of medications through inclusion of fixed-dose combination therapy on the national essential medicines list, as well as implementation of the drug revolving fund to optimize accessibility of blood pressure lowering medications (Figure 1). The full extent of impact in this domain remains contingent on ongoing cost analyses and subsequent developments in state and federal policy related to community insurance coverage of blood pressure lowering medications.

## Discussion

The improvements in hypertension treatment and control rates observed in the HTN Program are consistent with recorded improvements in other countries that have implemented HEARTS over a similar time frame and in similar settings [4]. As far as the authors are aware, the HTN Program is one of the largest and most effective programs of its kind in Africa and globally.

### Policy

A preliminary review detailing the early policy implications of the HTN Program has been published, but this report expands the evaluation of policy impact of the Program [21]. The continuous collaboration between the study team and governmental agencies such as the Nigerian Federal Ministry of Health, Federal Capital Territory Primary Healthcare Development Agency, Federal Capital Territory Department of Public Health, and local government area council public health leaders was instrumental in implementing the HTN Program and in translating results into policy action through sharing of data and findings. Improving health policy strengthens the multilevel impact of the program by providing a longer-term, government-supported investment in the reduction of hypertension burden. This program aligns with goals outlined in the National Multi-Sectoral Action Plan for the Prevention and Control of Non-Communicable Diseases, published in 2019 [22], which enhanced the willingness of policymakers to engage with the research team. The implementation of evidence-informed policies to improve hypertension service delivery is a long-term process, is inherently multilevel, and dependent on continuous stakeholder engagement, including among government agencies and officials.

### Capacity building

While not in the original TSBM model, capacity building is a domain that is often under-reported though not undervalued by those who directly benefit. The study team divided Capacity Building into three indicators that align with TSBM and distinguish different types of capacity building.

- *Training and Skills*

An integral component of the multilevel hypertension control program used in the HTN Program was the ability to manage hypertension at the PHC level by non-physician healthcare workers, including CHEWs. This is especially unique in the HTN Program as the program was able to use and empower CHEWs to manage hypertension, integrating with National Task Shifting and Task Sharing priorities to streamline access to care [16]. In-person training was provided to at least two CHEWs from each participating site [5]. Additionally, online virtual learning was conducted for over 1,000 healthcare workers across 12 PHCs [23]. Continuous training and supportive supervision were provided to non-physician healthcare workers for the duration of the HTN Program, which led to improved ability to evaluate and manage patients with hypertension.

- *Infrastructure*

The development of research infrastructure at the University of Abuja was essential in leading and operating a multilevel hypertension control program. As the central hub of the HTN Program, researchers and facilities at the University of Abuja gained skills and experience, increasing institutional capacity to conduct research through a new Cardiovascular Research Center at the University of Abuja Teaching Hospital, which opened a new research facility in 2024.

- *Research training and career development*

The HTN Program included several early-career investigators, and the implementation of the HTN Program supported a Fogarty-funded research training program (D43TW011976), as well as two career development awards (K23HL173684, K24HL175228), among other research and training awards. The HTN Program has served as a platform for early-stage investigators, postdoctoral research fellows, PhD candidates, and others to initiate work with the support and supervision of experienced researchers. Opportunities to develop research questions, refine skills, and demonstrate capacity to lead research teams build research competency and experience in those who will continue this and related research going forward.

Large scale studies like the HTN Program require extensive formative assessment. Results of these initial assessments often lead to changes in infrastructure, protocol, and training. In the HTN Program, PHCs not only needed training and supervision to diagnose and manage patients with hypertension, but they also needed training and supervision in medication stocking, equipment function and testing, and accurate record-keeping [24]. These updates or modifications that were required can be sustained once the active implementation period ends. Sustainment of hypertension services is another area of future research during the second phase of the program using routinely collected data that are linked and reported through the District Health Information System (DHIS-2).

### Community and public health

The integration of the HTN Program into PHCs, as well as the enhanced clinical capacity of healthcare workers, improved accessibility and reach of hypertension care services [7]. PHCs also served as community hubs to raise knowledge and awareness about hypertension among members in the community, not just patients enrolled in the HTN Program. In response to stakeholder feedback during formative evaluation, the study team sought to improve knowledge of hypertension among the general public through community awareness meetings [15]. From March 2020 to December 2022, over 300 meetings were held with over 6000 estimated attendees, helping to improve awareness of hypertension and hypertension services to improve the cascade of care (Ojo et al. under review).

### Clinical and medical

The HTN Program improved the quality of hypertension care to over 21,000 Nigerian adults, nearly half (46%) of whom had only primary school or less education (Ojji et al. under review). Direct clinical improvements in hypertension treatment and control, as well as the reductions in systolic and diastolic blood pressure, were key effectiveness outcomes. However, the improvement in quality of care, integration of diagnostic services, and potential to integrate, sustain, and spread hypertension evaluation and management across Nigeria’s six geopolitical zones during the program’s second phase are benefits that were not included in the original study plan. Additionally, the integration of hypertension care at the primary care level has laid the foundation for ongoing formative research on integrating diabetes care into PHCs in the FCT, which is also part of the second phase of this program. This integration of multimorbid care can improve the quality of primary healthcare for many Nigerians living with both conditions [20].

### Economic

While the program initially provided free medications to patients, the implementation of a drug revolving fund highlighted a sustainable financial strategy to increase accessibility of blood pressure lowering medicines. Additionally, the work done by the HTN Program study team was instrumental in placing key blood pressure-lowering medications on Nigeria’s essential medicines list, a necessary step to promote affordability of medications among hypertensive patients. Study participants who were eligible for community insurance from the Basic Healthcare Provision Fund and National Community Health Insurance Scheme were enrolled in some primary healthcare facilities after the implementation period, thereby lowering financial barriers to care.

### Strengths and limitations

The strengths of using the TSBM as a translational science impact framework range from its development at the Washington University Institute for Clinical and Translational Sciences and its breadth, depth, and flexibility in capturing non-traditional impact areas in a variety of research settings. As the TSBM gains favor among the translational science community, efforts are being made to create tools to systematically identify benefits within the TSBM framework and thus promote standardization [25]. The TSBM also serves as a resource for continuous, longitudinal impact identification, and using newly developed tools to revisit and systematically identify HTN Program benefits in the future can help compare benefits and data across different studies, including during future phases of the program.

Despite these strengths, there are also limitations when using the TSBM as an impact framework. Chief among these is the fact that there is no standardized metric that can quantify the magnitude of impact across benefits or domains. This lack of standardized, objective assessments makes comparing or evaluating the “importance” of different impacts, even within the same study, difficult given the subjective nature of the assessment. Data supporting demonstrated benefits are dependent on the design and process implemented to obtain the data, and so the strength of evidence can be variable. Additionally, contributions to policy changes are multifactorial, and the authors recognize the multitude of efforts made by various individuals and organizations to pass health promoting policies, of which the HTN Program was a contributor. The HTN Program team acknowledges that there may be inherent social desirability and reporting bias to identify and report on the impact of the HTN Program; however, the team believes the identified benefits are an accurate representation of its impact that would be corroborated by an independent assessment. We cite public reports related to impact across the TSBM domains to support our claims. Contemporaneous evaluation of impact likely improved the validity of the findings.

The observed and sustained improvements in hypertension treatment and control among the study population of over 21,000 patients are highly encouraging. The program has advanced toward national scale-up through implementation across Nigeria’s five other geopolitical zones in its second phase [26]. Implementation of the adapted HEARTS technical package in primary care was shown to be effective at reducing systolic and diastolic blood pressure and maintaining hypertension control rates greater than 50% for more than 18 months (Ojji et al. under review). While the primary goal of this research program was to improve cardiovascular service delivery, the impact of the HTN Program outside of its proposed primary and secondary outcomes should also be acknowledged.

## Conclusion

To our knowledge, few studies have applied the TSBM in LMIC settings. This manuscript seeks to address this gap by providing both a rationale and a concrete example of using and adapting the TSBM in global contexts. Using the TSBM to examine the impact of the HTN Program resulted in the identification of 33 benefits spread across five domains (Clinical, Community, Economic, Policy, and Capacity Building). Continuous monitoring of new developments and policies is necessary to determine and identify longer-term impact of the HTN Program, which has entered a second phase focused on sustainability of the initial gains in hypertension care, national scale-up of hypertensive services, and integration of diabetes management in primary care in the FCT. As one of the largest facility-based hypertension treatment program in sub-Saharan Africa [5], the HTN Program hopes to provide a pathway and framework for similar models to be applied in similar settings. Future research to evaluate the impact will be included to determine the extent to which the program continues to achieve and exceed its goals.

## Table 1. Long description

The table compares structural components of multilevel hypertension control programs across three models: Kaiser Permanente Northern California, World Health Organization HEARTS, and Hypertension Treatment in Nigeria. It has seven rows and four columns. The columns are labeled Component, Kaiser Permanente Northern California model, World Health Organization HEARTS model, and Hypertension Treatment in Nigeria program. The rows are labeled with different components: Patient registry/empanelment, Performance and quality reporting, Simplified treatment protocols and patient care, Supply chain (including fixed-dose combination prioritization and access to essential medicines), Team-based care, and Supportive supervision. Each cell contains specific details about how each component is addressed in the three models. For example, under Patient registry/empanelment, Kaiser Permanente Northern California model uses hypertension patient registration using baseline data, World Health Organization HEARTS model uses hypertension patient registration, and Hypertension Treatment in Nigeria program uses hypertension patient registration and longitudinal empanelment. The table provides a detailed comparison of the approaches and strategies used in each model for managing hypertension.

Navigate back to Table 1..
